# Accelerated proteomic visualization of individual predatory venoms of *Conus purpurascens* reveals separately evolved predation-evoked venom cabals

**DOI:** 10.1038/s41598-017-17422-x

**Published:** 2018-01-10

**Authors:** S. W. A. Himaya, Frank Marí, Richard J. Lewis

**Affiliations:** 1 IMB Centre for Pain Research, Institute for Molecular Bioscience, The University of Queensland, Queensland, 4072 Australia; 2 Marine Biochemical Sciences, Chemical Sciences Division, National Institute of Standards and Technology, 331 Fort Johnson Road, Charleston, SC 29412 USA

## Abstract

Cone snail venoms have separately evolved for predation and defense. Despite remarkable inter- and intra-species variability, defined sets of synergistic venom peptides (cabals) are considered essential for prey capture by cone snails. To better understand the role of predatory cabals in cone snails, we used a high-throughput proteomic data mining and visualisation approach. Using this approach, the relationship between the predatory venom peptides from nine *C. purpurascens* was systematically analysed. Surprisingly, potentially synergistic levels of κ-PVIIA and δ-PVIA were only identified in five of nine specimens. In contrast, the remaining four specimens lacked significant levels of these known excitotoxins and instead contained high levels of the muscle nAChR blockers ψ-PIIIE and αA-PIVA. Interestingly, one of nine specimens expressed both cabals, suggesting that these sub-groups might represent inter-breeding sub-species of *C. purpurascens*. High throughput cluster analysis also revealed these two cabals clustered with distinct groups of venom peptides that are presently uncharacterised. This is the first report showing that the cone snails of the same species can deploy two separate and distinct predatory cabals for prey capture and shows that the cabals deployed by this species can be more complex than presently realized. Our semi-automated proteomic analysis facilitates the deconvolution of complex venoms to identify co-evolved families of peptides and help unravel their evolutionary relationships in complex venoms.

## Introduction

Cone snails are venomous marine molluscs that hunt fish, molluscs and worms depending on their prey preference. The major components of *Conus* venom are small structured peptides (conopeptides or conotoxins) that are injected using a hollow, barbed radula tooth for prey capture or defense^1^. There are ~850 species of cone snails identified^2^ with each expressing many thousands of unique peptides^3–6^ that selectively target a diverse range of voltage- and ligand gated ion channels, transporters and G-protein couple receptors^7^. Given their high potency and isoform selectivity, cone snail venom peptides provide a natural reservoir of potential drug leads^7–9^. The ability of cone snails to switch between separately evolved predatory and defensive venom regimes appears to underpin this remarkable structural and functional diversity. However, it remains unclear what contributes to their remarkable inter-and intra-species variability^6,10–13^.

To overcome the limitations of traditional low throughput and less sensitive peptide identification methods, high throughput approaches such as integrative transcriptomics and proteomics are starting to be applied^4–6^. However, the “omics” data explosion requires complementary rapid data analysis and interpretation tools to make sense of embedded relationships. Statistical methods such as Principal Component Variable Grouping and Hierarchical clustering allows assignment of a large number of variables to a smaller number of groups for enhanced visualization^14,15^. However, this approach has not been applied to analyse the complex proteomic data from venoms. To overcome this constraint, we developed an accelerated approach to proteomic data analysis and applied it to better understand the prey capture strategy and venom complexity of nine *C. purpurascens*.

*C. purpurascens* is an Eastern Pacific fish hunting cone snail that uses a hook and line strategy to catch fish^16^. By chemically, pharmacologically and behaviorally characterizing the pooled predatory venom of *C. purpurascens*, two cabals were identified to be associated with rigid paralysis (lightning-strike cabal) and flaccid paralysis (motor cabal) of prey^16–21^. The lightning-strike cabal of *C. purpurascens* was shown to comprise two excitatory peptides, κ-PVIIA and δ-PVIA, which inhibit potassium channels and delay inactivation of sodium channels, respectively^16–18^. In contrast, *C. purpurascens* motor cabal comprised inhibitory peptides including µ-PIIIA, αA-PIVA, α-PIB that inhibit sodium channels and nicotinic acetylcholine receptors^22–24^.

Given the surprising individual variability observed in *C. purpurascens* injected predatory venom^11,13^, we were interested in determining if the cabals identified previously in their pooled predatory venom were present in individual *C. purpurascens*. In this study, injected predatory venom from nine *C. purpurascens* was analyzed by LC-ESI-triple-TOF-MS to determine the prey capture strategy used by each specimen. Our analysis revealed a remarkable variation in the prey capture cabals deployed, with most individuals using either the lightning strike or the motor cabal and only one specimen deploying both cabals. Similar results were obtained using both manual and a new semi-automated peptide measurement approach, validating the use of this approach to accelerate the deconvolution of complex proteomes.

## Results and Discussion

The injected venom collected from nine specimens of *C. purpurascens* was analyzed using nanoflow LC-ESI-TripleTOF-MS to identify the conopeptide profile of each specimen, as previously described^13^. While duplicate milkings from each individual were the same, except for one “blank injection”^25^, the venom obtained from the first milking was used for analysis. Using a manual approach, we were able to assign predatory venom cabal profiles across these nine specimens. To broaden and accelerate this approach, we developed an accelerated *de novo* approach that revealed the separate motor and lightning strike cabals have coevolved with distinct clusters of currently uncharacterized conotoxins.

### Variability of predatory venom profiles across nine *C. purpurascens*

**Figure 1:**
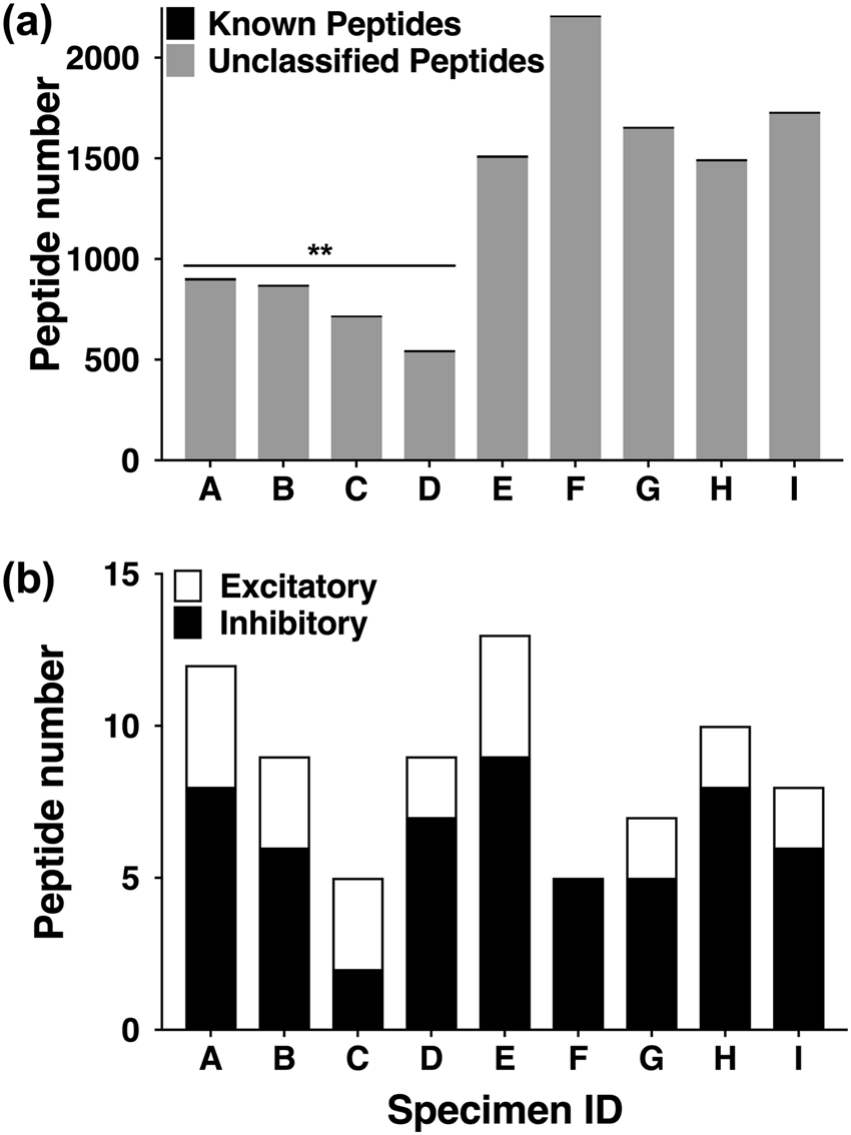
The number of masses detected in the injected predatory venom of *C. purpurascens*. (**a**) The number of known and unknown masses in the injected predatory venom of each specimen. (**b**) The contribution of known excitotoxins and neuromuscular blockers to the venom of each specimen. Two μL of the venom from each specimen was analysed with Sciex TripleTOF 5600 instrument. The masses were identified using the Analyst™1.6 program (Sciex) and were pre- processed to detect and remove duplicates, Na^+^ and K^+^ adducts using mass processing tools of ConoServer. The resulting number of unique masses from each specimen is graphically represented. A significant difference (P < 0.01) in the peptide number between specimens A to D and E to I was found with unpaired t-test.

A comparison of the LC-MS total ion chromatograms revealed significant inter-specimen variability (Supplementary Fig. S1). To gain an initial understanding of the complexity of individual predatory venoms, the total peptide count in each specimen was compared and the visual representation indicates the large number of (>99%) uncharacterized peptides found in each venom (Fig. 1). Specimens E, F, G and I were most complex (>1500 peptides each), while specimens B, C and D were least complex (<900 peptides each). Specimens A–E contained prominent late eluting (17–19 min) hydrophobic peptides, while specimens F–I lacked these hydrophobic peptides and instead contained early eluting (3–5 min) hydrophilic peaks (Supplementary Fig. S1). Only one individual (specimen E) contained both early and late eluting peptides. Thus, it is evident that individual *C. purpurascens* predatory venoms provide fingerprints that presumably reflect divergent responses to selection pressures including prey availability, competition, climate, gender and environmental factors^26^. This venom peptide divergence is presumably driven by gene duplication, recombination and hypermutation events^27^, transcriptomic messiness^5^ and variable peptide processing^4^.

### Variability of the lightning-strike-cabal peptides across nine *C. purpurascens*

Given the variability seen across the nine specimens, we systematically identified all previously identified peptides contributing to the lightning-strike cabal of *C. purpurascens* and their expression intensities tabulated (Table 1). The LC-MS/MS support for known peptides are shown in Supplementary Fig. S2. Surprisingly, the presence of lighting strike cabal peptides δ-conotoxin PVIA and κ-conotoxin PVIIA varied across the nine specimens, despite their reported pivotal role in prey capture by *C. purpurascens* ^16–21^. Only specimens A–E produced both PVIA and PVIIA, while PVIA or its known post translational modification (PTM) variants^13^ were absent in specimens F–I, and only very low levels of PVIIA (<0.1% relative intensity) were detected in these specimens. A ProteinPilot search on the tryptic digested LC-MS/MS chromatograms of specimens F to I did not reveal any novel sequences with close similarity to PVIA or PVIIA. Interestingly, PVIA is either expressed at high levels or absent, indicating that PVIA is a key component of the prey capture venom only in a subset of specimens.

**Table 1:** Relative expression levels of the previously identified peptides in the predatory injected venom of *C. purpurascens* across nine specimens detected in ESI-triple-TOF-MS*.

Specimen	A	B	C	D	E	F	G	H	I
**Lightning Strike cabal peptides**									
δ-PVIA	++++	++++	++++	++++	++++	−	−	−	−
κ-PVIIA	+++	+	++	+++	++	−	+	+	+
**KappaA peptides**									
κA-PIVE	+	++++	−	−	+++	−	−	+	+
κA-PIVF	++++	−	+	−	+	−	+	−	−
**Motor cabal peptides**									
α-PIA	+	+	−	+	+++	+	−	+	−
α-PIB	−	−	−	−	++++	−	+	+	−
αA-PIVA	+	++	+	+	++	++++	−	++++	++++
μ-PIIIA	+	+	−	+	+	+	−	+	+
ψ-PIIIE	+	+	−	+	++++	++++	++++	++++	++++
ψ-PIIIF	+	−	−	−	+	+	−	+	+
**Unclassified peptides**									
PVA	+	−	+	+	++	+++	++	+	++
p6a	+++	+++	−	+++	++	−	+	+	−
p6b	+	++	−	++	++	−	+	−	+

*++++ Relative intensity more than 50, +++ relative intensity between 50 and 10, ++ relative intensity between 10 and 1, + relative intensity below 1, – not detected.

κA-conotoxins that induce hyperactivity and spastic paralysis in fish and mice have been widely identified from hook- and line fish hunters^6,28,29^. Given their excitatory effects, κA-conotoxins are considered a component of the lightning strike cabal^19,20^, with the *pionoconus clade* (*C. catus, C. striatus, C. consors)* using glycosylated κA conotoxins as their main excitatory component^6,30^. Most κA-conotoxins are O-glycosylated^31^ although the shorter excitatory κA -PIVE and κA -PIVF from *C. purpurascens* are not glycosylated^28^. However, despite the absence of δ- and κ-conotoxins in the venoms of some *C*. *purpurascens* specimens, κA-conotoxin were found in specimens A, B and E (Table 1). Thus overall, four of nine specimens contained no detectable lightning strike cabal peptides but instead contained significant levels of motor cabal peptides, as discussed below.

### Variability of the motor cabal peptides across nine *C. purpurascens*

Complimentary to the expression levels of excitatory peptides, motor cabal peptides α-PIA, α-PIB, αA-PIVA, μ-PIIIA, ψ-PIIIE and ψ-PIIIF were present in specimens E–I (Table 1), with only specimen E containing both excitatory and inhibitory cabal peptides explaining its complex LC-MS profile. Among the known inhibitory peptides, ψ-conotoxin PIIIE was often most abundant peptide (>50% of the relative intensity in E–I). PIIIE is a non-competitive inhibitor of skeletal nAChR that causes flaccid paralysis when injected into teleost and gold fish^18,32^. PIIIE is structurally related to the μ-conotoxins that inhibit Na^+^ channels^7^ and our finding suggests it plays an important role in the predatory venom of *C. purpurascens*. Another muscle nAChR blocker αA-PIVA peptide^33^, was found in all specimens except specimen G. PIVA was a dominant component in specimens F, H and I, while the two remaining known α-conotoxins α-PIA and α-PIB were predominant only in specimen E (Table 1). Finally, the previously identified T superfamily peptide PVA was found in all the specimens except specimen B, while two O1 superfamily peptides p6a and p6b were prominent in specimens A, C, D and E (Table 1). The pharmacological targets of late eluting p6a and p6b are not confirmed yet.

All specimens were collected from a same location at the same time (Pacific Shores of Costa Rica) and therefore the differences of venom composition cannot be attributed to the differences in climate, food availability and other environmental factors. Thus, it appears that *C. purpurascens* have evolved as two sub-species that utilize either excitatory or inhibitory cabals and associated peptides, with only one of nine specimens showing the ability to utilize both cabals for predation.

### Semi-automated proteome mining and visualisation

**Figure 2:**
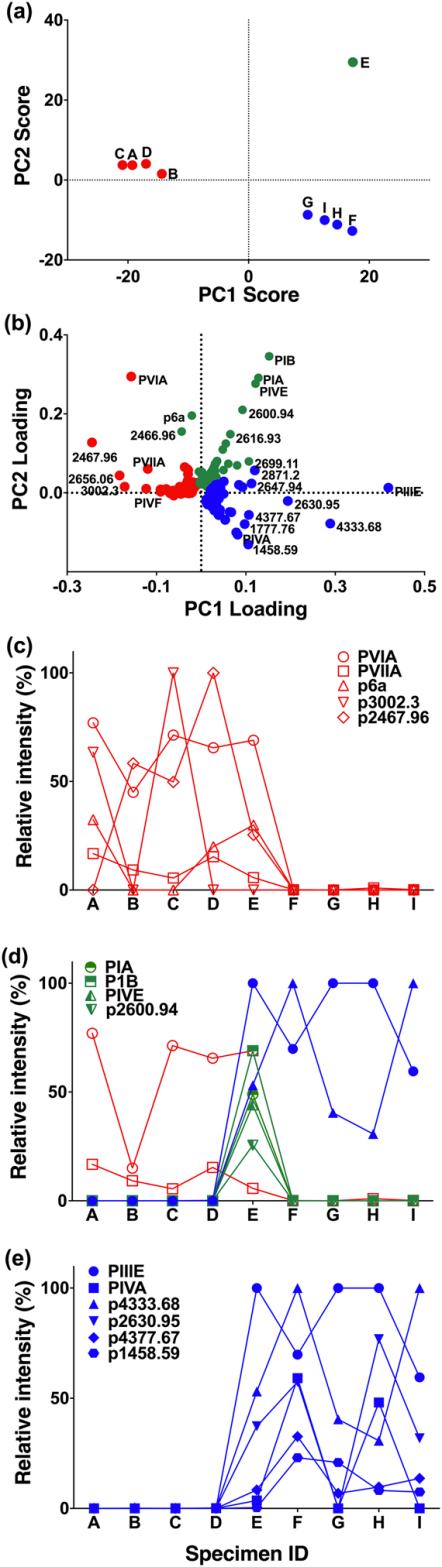
Clustering pattern of nine *C. purpurascens* specimens and grouping of the peptides in each cluster. (**a**) Scores plot generated in the PCA analysis of the LC-ESI Triple TOF-MS data of each predatory venom. The specimens with similar venom profiles are clustered together while different ones are well separated. (Scores for PC1 79.9% Vs PC2 14.6%, Pareto Scaling). (**b**) The Loading plot shows contribution of 1000 peptides shared across 9 specimens to the venom variability among the clusters. The expression patterns of the dominant unique peptides grouped into each group; group 1 (**c**), 2 (**d**) and the hybrid of group 1 and 2 (**e**).

The manual analysis described above identified variable cabal profiles, although the complexity of each venom profile restricted this analysis to dominant and known conotoxins. To accelerate and broaden this approach, we used Markerview1.3^TM^ (Sciex) in combination with statistical tools to directly analyse and visualize LC-MS data to find peptide ions in complex data sets^34,35^. In the current study the data matrix generated in the Markerview1.3^TM^ software, was further analysed using PCA (principal component analysis), an unsupervised multivariate statistical analysis method, to compare data across multiple samples to reveal groupings among data sets^35–37^. The sample groupings can be clearly seen in the Scores plot (Fig. 2a) and the Loading plot (Fig. 2b) provides insight into variable peptides that lead to the variation across samples. Specimens A–D (group 1) expressed lightning-strike cabal and related excitatory conotoxins along with a distinctive set of novel conotoxins but no identified motor cabal peptides (Fig. 2c). In contrast, specimens F–I (group 2) expressed motor cabal peptides including nAChR blockers and a cluster of novel peptides (Fig. 2e). Interestingly specimen E clustered separately (hybrid of group 1 and 2) and contained significant levels of both group 1 and 2 conotoxins along with unique known (PIA, PIB and PIVE) and novel peptides (p2600.9) (Fig. 2d).

**Figure 3:**
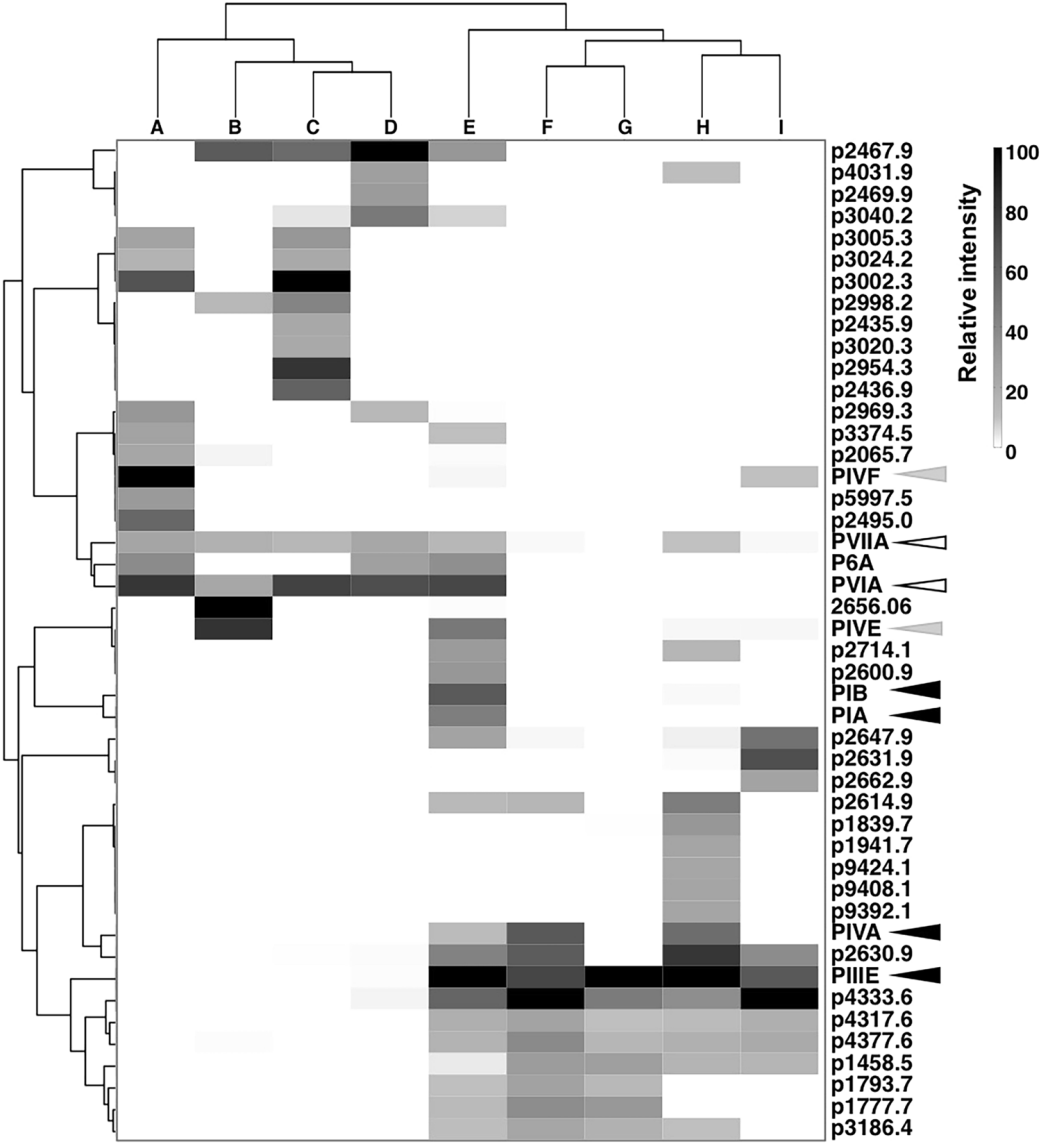
Visualisation of the injected venom profiles of *C. purpurascens*. The heatmap matrix displays the relative expression levels of previously identified and abundantly expressed novel peptides in the pooled proteome. The grouping of the specimens is shown in the top dendrogram. The hierarchical clustering of the peptides (distance correlation, average linkage method) reveals a unique peptide expression patterns in each group. Symbols key; dark arrows indicate motor cabal peptides, white arrows indicate lightning strike cabal peptides and grey arrows indicate excitatory kA peptides.

The top 50 highly expressed peptides in the proteome of 9 specimens was used to generate a hierarchical cluster-gram across the peptides and specimens. A heatmap was generated to visualise the peptide expression patterns in each specimen (Fig. 3). This approach revealed that specimens A to D and specimens F to I clustered separately, whereas specimen E had features that overlapped both clusters. A similar pattern was obtained following manual analysis of these individual venom profiles. This pattern was also clearly seen in the heatmaps generated using top 100 and 1000 and 3207 (total number of unique peptides) peptides of the total proteome (Supplementary Fig. S3), indicating this distinct compositional variability across to the minor components. Thus, *C. purpurascens* collected in the same region of Costa Rica have developed two separate and distinct prey capture strategies, one producing tetanic paralysis through excitatory actions on Na^+^ and K^+^ channels and one producing flaccid paralysis through inhibitory actions on Na^+^ channels and nAChRs.

PCA analysis and clustering revealed a large number of uncharacterised peptides have co-evolved with either excitatory cabal or inhibitory cabal (Fig. 3). For example, the peptide p4333.6 eluting at 5.3 min (Supplementary Fig. S3) was abundant (>45% relative intensity) only in specimens E–I expressing motor cabal conotoxins and it can be predicted to contribute to the motor cabal. In contrast, peptide p3002.2 was the dominant (>63%) conotoxin in specimens A and C, while peptide p2467.9 (>40%) in specimens B–E. Apart from these dominating novel peptides there were large number of novel masses clustering with either group (Figs 2 and 3). Thus, our clustering approach can identify uncharacterized conotoxins likely to contribute to either the excitatory or inhibitory venom cabal of *C. purpurascens*, providing a rational approach for the identification of function in novel conotoxins.

Conotoxins share structural and sequence homology^7^ that facilitates the prediction of pharmacology of related novel peptides. A ProteinPilot search of tryptic digested LC/MS-MS chromatograms revealed all novel masses in specimens F to I, including p4333.6 or p2467.9, had no sequence similarity to PVIA, PVIIA or other known excitatory conotoxins, confirming the absence of variants that might replace known excitatory conotoxins in these predatory venom. However, PVIA and PVIIA was found at the transcriptomic level of *C. purpurascens* specimen lacking the lightning strike cabal peptides in the predatory venom (unpublished work, Frank Mari). Seemingly, the expression of conotoxins is regulated translationally or post-translationally, potentially at the level of precursor trafficking and/or processing.

Given only one of nine specimens contained both excitatory and inhibitory cabal peptides indicates that snails possessing both cabals do not have an evolutionary advantage. Prey preference is thought to be a major driver of venom composition and diversification patterns, with natural selection shaping the venom repertoires of species to be more effective at targeting their preferred prey^38^. Reflecting their recent evolution, the estimated rates of gene duplication and non-synonymous substitutions for conotoxin genes are the highest across metazoans. These extraordinary rates of molecular diversification promote divergence rather than convergence in venom composition^39^ and likely contribute to speciation in Conidae.

## Conclusions

Overall, these results reveal two major prey capture cabals employed by individual specimens of *C. purpurascens* with only one specimen having both cabals present in the injected predatory venom. However, at this time it is not possible to establish if specimen E represents an ancestral form or arose from interbreeding between two sub-species of *C. purpurascens*. Opposing the established theory on the predatory venom of hook-and-line fish hunting cone snails, this study for the first time reveals the lightning strike and motor cabals are interchangeable cabals, with neither being essential for effective prey capture by *C. purpurascens*. Using a validated integrated computational approach, we were able to rapidly unravel patterns and relationships between venom samples, revealing clusters of apparently co-evolved venom peptides associated with known excitatory cabal components that may help unravel novel bioactive peptides and their evolutionary links.

## Methods

### Specimen Collection and Venom Sample Preparation

Injected venom from nine *C. purpurascens* (Pacific shores of Costa Rica) was collected in predatory mode as previously described^13^. The cone snails were milked once a week and the predatory venom supernatant stored at −80 °C prior to LC-MS analysis.

### Mass spectrometry analysis of injected venom

Nano flow liquid chromatography-electrospray mass spectrometry (LC–ESI–MS) was performed on a TripleTOF 5600, a hybrid quadruple TOF MS equipped with a DuoSpray ionization source coupled to a Shimadzu 30 series HPLC system (Sciex^TM^, Framingham, MA). LC separation was performed on a Thermo C18 column (4.6 × 150 mm) eluted with a 1.3% B (90% acetonitrile/0.1% formic acid) over 20 min at a flow rate of 0.3 mL min^−1^. Samples corresponding to a single milking of injected venom (~2 μL) was injected into the LC-ESI-MS and scanned over the mass range *m/z* 300–2000 to obtain a mass list of venom peptides. LC-ESI-MS on replicate milkings of the same specimens confirmed that each specimen produces a unique venom.

For the LC–ESI–MS/MS analysis was performed in both TripleTOF 5600 (Sciex^TM^, Framingham, MA) system and Thermo Fisher Scientific Orbitrap Fusion^TM^ Lumos^TM^ Tribrid^TM^ Mass Spectrometer operating in positive mode coupled to a Thermo Fisher Scientific UltiMate 3000 UHPLC system. Aliquots of venom were lyophilized and subjected to reduction and alkylation using the previously described triethylphosphine/iodoethanol protocol^4^. Sigma proteomics sequencing grade trypsin was used for enzyme digestion of reduced and alkylated peptides, as described^5^. Information-dependent acquisition was performed on the reduced, reduced/alkylated, and enzymatically digested venom samples. The Orbitrap LC- MS/MS system was operated in DDA mode and the MS/MS Data analysis was carried out using the Peaks v8.0 software (Bioinformatic Solutions Inc., Dublin, Ireland).

ProteinPilot^TM^ 4.0 (Sciex^TM^, Framingham, MA) software was used for peak list generation and sequence identification by searching the LC−ESI−MS/MS spectra generated using TripleTOF 5600 system against the known *C. purpurascens* peptide sequences using previously described parameters^4–6^.

### Proteomic data processing

Mass spectrometric data was first subjected to LC-ESI-MS reconstruction using the biotool “LCMS reconstruct” in Analyst^TM^ (version 1.6) software (Sciex^TM^, Framingham, MA) with the mass range set between 1000–10000 Da, mass tolerance set to 0.2 Da, and S/N threshold of 10. The reconstructed MS data was then processed further to remove Na^+^ and K^+^ adducts and remove duplicate masses using the embedded tools in ConoServer (http://www.conoserver.org)^40^. The resulting peptide mass lists of each individual was searched against the known conotoxin sequence masses of *C. purpurascens* (Conoserver) using the “Compare mass lists” tool with a precision level set at 0.25 Da.

### Rapid proteomic analysis pipeline

The processed LC/MS mass lists containing the monoisotopic mass, retention time and relative intensity was imported into the MarkerView^TM^ (version 1.3.1) software (Sciex^TM^, Framingham, MA) to generate the proteome matrix, a list of all peptide masses and their relative intensities across 9 specimens. Relative intensities (percentage of maximum) were generated as a percentage of the most abundant peptide in each individual venom using Analyst^TM^ (version 1.6) software. The software locates unique masses/peaks in the list/spectra using spectral mass/peak finding algorithm followed by aligning the masses according to the retention time and filtering the background ions. Data alignment algorithms in Markerview1.3^TM^ software compensates for minor variations in both mass and retention time values, ensuring that identical compounds across samples are accurately compared to one another. The parameters for peak finding, alignment and filtering were set as: noise threshold 10, minimum spectral peak width 5 ppm, maximum RT peak width at 100 scans, retention time tolerance a 0.5 min, mass tolerance at 25 ppm and maximum number of peaks at 6000. The generated peak list data containing all unique masses and their relative abundancies across all nine specimens was used as the main data matrix for further analysis. This data matrix was used to perform Principal component analysis (PCA) to visualize the clustering patterns specimens in a Scores plot. The Loading plot revealed the peptides that contributes to the clustering patterns. Then the most abundant peptides (top 50) of the data matrix was used to generate the hierarchical cluster-gram (correlation distance, complete linkage method) across the peptides and nine specimens using Clustvis webtool^41^. The relative intensity heat map complementary to the hierarchical dendrograms (across peptides and specimens) was generated in GraphPad Prism (version 7.0c).

### Data Availability

The datasets generated during and/or analysed during the current study are not publicly available but are available from the corresponding author on reasonable request.

### Disclaimer

Certain commercial equipment, instruments, or materials are identified in this paper in order to specify the experimental procedure adequately. Such identification is not intended to imply recommendation or endorsement by the National Institute of Standards and Technology, nor is it intended to imply that the materials or equipment identified are necessarily the best available for the purpose.

## Electronic supplementary material

Supplementary Figs 1–3
