# Supplementary material for: Accelerated proteomic visualization of individual predatory venoms of Conus purpurascens reveals separately evolved predation-evoked venom cabals
Source: Sci Rep. 2018 Jan 10;8:330. doi: 10.1038/s41598-017-17422-x (PMC5762640; doi:10.1038/s41598-017-17422-x)
Supplement: Supplementary file 1 — Supplementary Figs 1–3 [file 41598_2017_17422_MOESM1_ESM.pdf]

Accelerated proteomic visualization of individual predatory venoms of *Conus purpurascens* reveals separately evolved predation-evoked venom cabals

S.W.A. Himaya[1], Frank Marí[2], Richard J. Lewis[1,*]

[1]IMB Centre for Pain Research, Institute for Molecular Bioscience, The University of Queensland, Queensland 4072, Australia

[2]Marine Biochemical Sciences, Chemical Sciences Division, National Institute of Standards and Technology, 331 Fort Johnson Road, Charleston, SC 29412, USA

[*]Email: r.lewis@imb.uq.edu.au. Phone: +61 7 3346-2984. Fax: +61 7 3346-2101

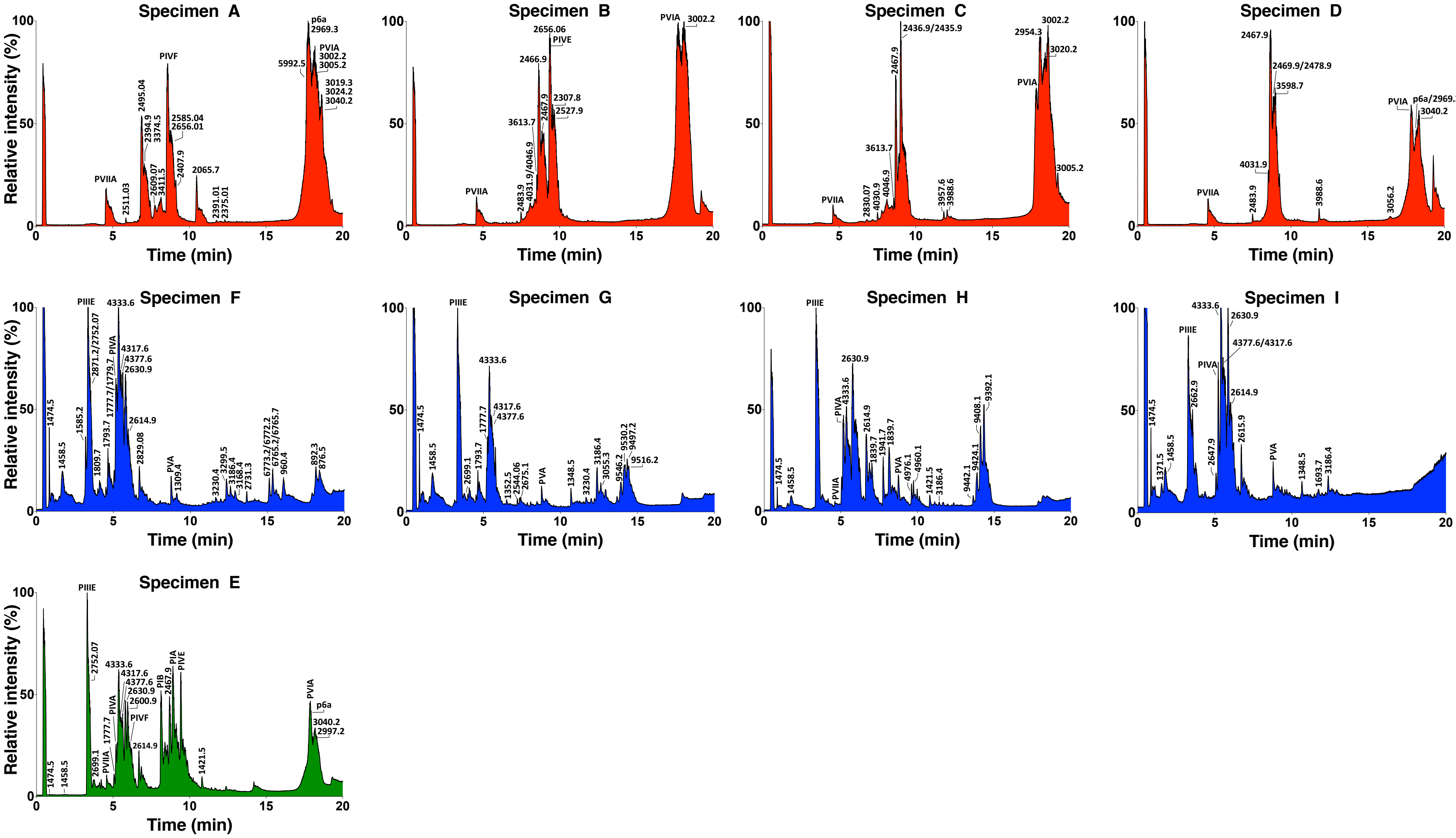

Figure S1: LC-ESI Triple TOF-MS total ion current chromatogram of the injected predatory venom from nine individual *C. purpurascens*. LC-MS run on the TripleTOF 5600 System revealed the complexity of the venom.

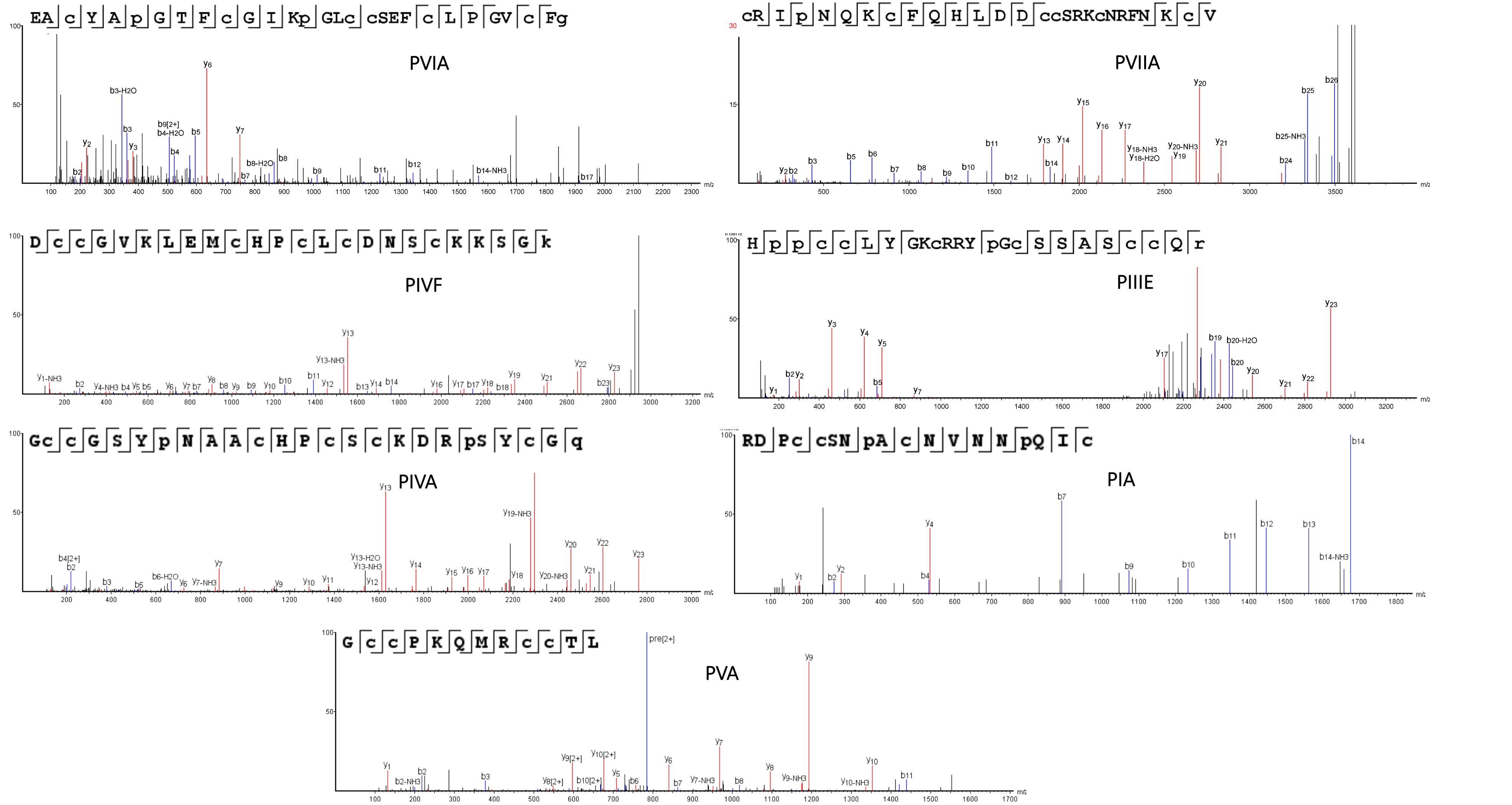

Figure S2: LC-MS/MS interpretations of the known peptides abundantly present in *C. purpurascens.* Thermo Fisher Scientific Orbitrap Fusion™ Lumos™ Tribrid™ Mass Spectrometer operated in DDA mode was used for data acquisition and MS/MS Data analysis was carried out using the Peaks v8.0 software.

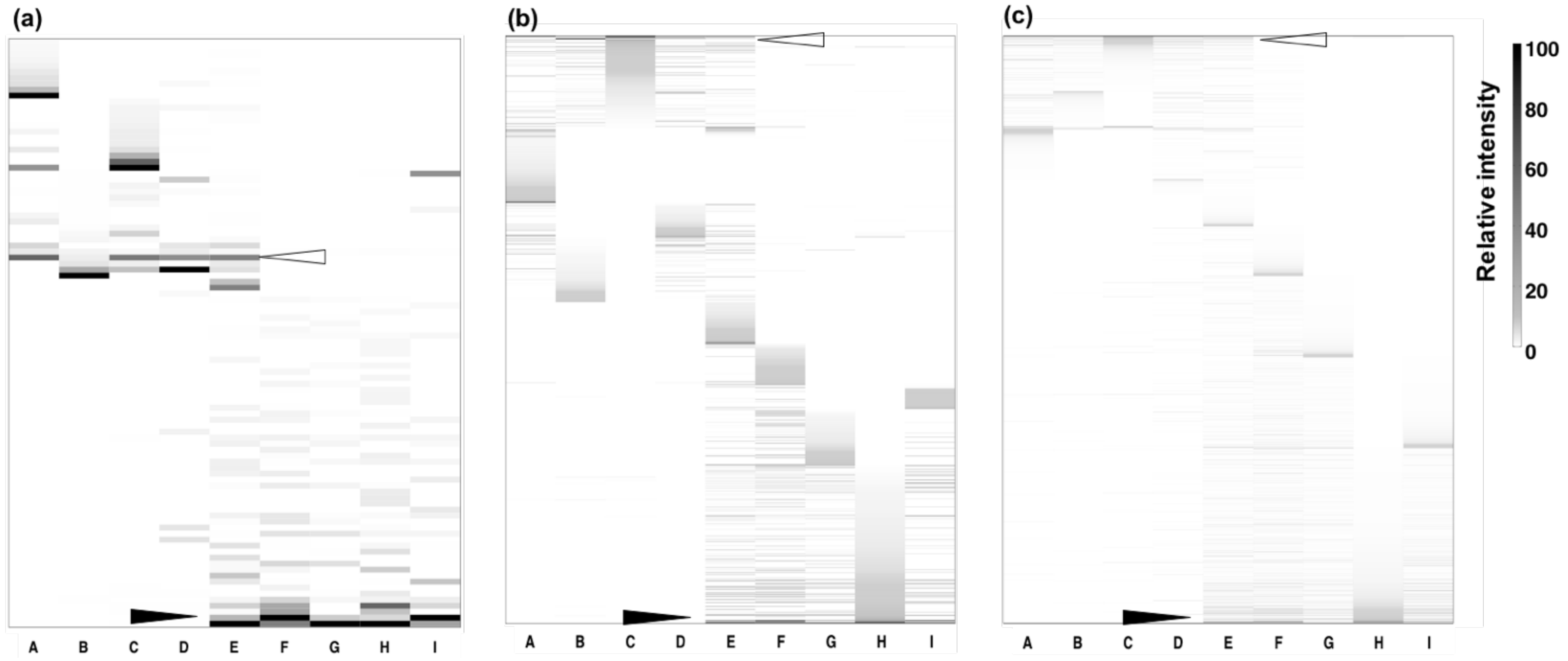

Figure S3: The heatmap matrices of relative expression levels of top 100 (a), 1000 (b) and the total pooled peptidome of 3207 peptides (c). The distinct peptide expression pattern is clearly extends into the minor components of the venom profiles of each specimen.
